# Janus kinase inhibitors for the therapy of atopic dermatitis

**DOI:** 10.5414/ALX02272E

**Published:** 2021-08-27

**Authors:** Stephan Traidl, Sina Freimooser, Thomas Werfel

**Affiliations:** Department of Dermatology, Allergology and Venereology, Hannover Medical School, Hannover, Germany

**Keywords:** atopic dermatitis, JAK inhibitors, baricitinib, cerdulatinib, delgocitinib, ruxolitinib, ­tofacitinib, upadacitinib

## Abstract

The JAK-STAT pathway is involved in the signaling of multiple cytokines driving cutaneous inflammation in atopic dermatitis (AD). Janus kinase (JAK) inhibitors target individual receptor-associated kinases, thereby preventing the mediation of inflammatory signals. Several JAK inhibitors with varying mechanism of action, potency, and safety represent potential therapeutic options for AD in both topical and systemic application. The JAK1/2 selective JAK inhibitor baricitinib was the first substance from this class of drugs approved by the EMA for the systemic oral treatment of AD. The clinical development program of the JAK1 selective inhibitors upadacitinib and abrocitinib is finalized with positive results for AD. The PAN-JAK inhibitor delgocitinib was the first substance being approved for the treatment of AD (in Japan). This review article covers the rising data on investigational and approved JAK inhibitors in the context of the treatment of AD.

## Introduction

Atopic dermatitis (AD) is one of the most common chronic inflammatory skin diseases affecting about 20% of children and up to 7% of adults [1, 2] Characterized by pruritus as a cardinal symptom, concomitant sleep disturbances and psychiatric diseases as comorbidity, there is a significant impairment of everyday life, especially in moderate-to-severe AD. Considering the lost healthy life years (DALY = disability-adjusted life years), AD represents the skin disease with the strongest impairment of life expectancy in good health [3].

The pathophysiology of AD involves a complex interplay of genetic predisposition, skin barrier defects such as increased transepidermal water loss (TEWL) and impaired lipid composition, environmental factors such as aeroallergens, and associated inflammation [4]. The latter is characterized by Th2 polarization with associated cytokines IL4, IL-5, IL-13, and IL-31, accompanied by Th17 and Th22 cells and, in chronic lesions, Th1 cells as well. Inflammation is not only characterized by T cells, but also by cytokines of keratinocytes, such as TSLP (= thymic stromal lymphopoietin) and IL-17C, cells of the innate immune system, such as ILC2 (= innate lymphoid cells type 2), and dendritic cells.

A survey among dermatologists in German-speaking countries****recently showed that they treat about 200 AD patients per year, of whom about 25% are moderately to severely affected. The main problems in the care of these patients were identified as the topical basic therapy as well as the supply with biologics, the latter being particularly affected by economic influences. The therapy of AD currently includes the basic therapy with an emollient. When necessary, topical glucocorticosteroids and calcineurin inhibitors are used. The phosphodiesterase 4 (PDE 4) inhibitor crisaborole, which is available in the USA, is not available in Europe, so that alternatives to the topical anti-inflammatory therapies mentioned are currently lacking. With the approval of dupilumab, a monoclonal IgG4 antibody against the IL-4 receptor alpha subunit, a new era in the treatment of moderate-to-severe AD was ushered in 2017 [5]. With the approval of baricitinib in late 2020, a Janus kinase (JAK) inhibitor, the first oral treatment option besides ciclosporin has been approved.

## Basis of JAK inhibition

JAKs represent a family of receptor-associated kinases. The name “Janus kinase” is based on the name of the Roman god “Janus”, the god of the beginning and the end, who is mostly depicted as two-headed. Here, parallels to the two adjacent kinase domains of JAKs become apparent.

**Figure 1.**
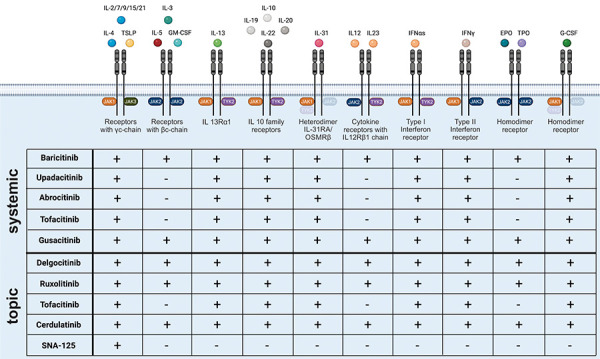
Cytokine receptors and their associated Janus kinases (JAKs). The different cytokines and growth factors bind to receptors associated with different JAKs. The different JAK inhibitors inhibit different JAKs, resulting in a diverse spectrum of activity. GM-CSF = granulocyte-macrophage colony stimulating factor; EPO = erythropoietin; IFN = interferon; TPO = thrombopoietin; TSLP = thymic stromal lympho­poietin.

Four different JAKs can be distinguished in mammals: JAK1, JAK2, JAK3, and TYK (= tyrosine kinase) 2. The function of JAKs is closely coupled to the cytoplasmic STAT (signal transducer and activator of transcription) transcription factors, of which seven are currently described (STAT1, STAT2, STAT3, STAT4, STAT5A/5B, and STAT6) [6]. Due to this interaction, it is also referred to as the “JAK-STAT signaling pathway”. This pathway mediates extracellular signals to the nucleus intracellularly and induces transcription of a wide variety of genes. The binding of molecules to the corresponding receptor leads to the activation of the corresponding JAKs and subsequently to the phosphorylation of tyrosine in the receptor. Binding of STATs is enabled, which are also phosphorylated by the JAKs. The activated STATs subsequently induce transcription of various genes in the nucleus [7]. Looking at the JAK-STAT signaling pathway in different Th cells, it is apparent that TYK2, JAK2, and STAT4, which are components of the IL-12 pathway, are essential for Th1 cell differentiation, whereas JAK1, JAK3, and STAT6, which are components of the IL-4 pathway, are critical for Th2 differentiation [6]. However, JAK2 also plays a critical role in the activation of the Th2-associated IL-5 receptor. Furthermore, STAT3 and STAT 5 play a role in the IL-4 signaling pathway [8]. JAKs are collectively involved in the signal transduction of >50 cytokines and growth factors [9]. Signaling of several cytokines that have been shown to have pathophysiological relevance in AD are also mediated via JAK-associated receptors (Figure 1), making JAKs potential therapeutic targets.

## JAK inhibitors

The role of JAKs in the pathophysiology of diseases was first demonstrated in 1995 with the identification of a loss-of-function mutation in JAK3 in severe combined immunodeficiency (SCID) [10, 11]. In 2005, gain-of-function mutations were described in myeloproliferative disorders such as polycythemia vera [12, 13]. The impetus provided by these discoveries led to the identification of the role of JAKs in a wide variety of diseases. Regarding inflammatory skin diseases, a contribution to the pathophysiology has been shown for AD, psoriasis, lupus erythematosus, alopecia areata, pyoderma gangrenosum, graft-versus-host disease, and hyper-IgE syndrome [14, 15].

A number of JAK inhibitors have been developed in the course. The selectivities given for the substances concerned are to be regarded as relative in principle, but generally apply to the doses used therapeutically.

Ruxolitinib, a JAK1 and JAK2 inhibitor, was the first JAK inhibitor approved in 2011 for the indication of primary and secondary myelofibrosis [16]. In the same year, the U.S. Food and Drug Administration (FDA) approved a second JAK inhibitor: tofacitinib (JAK1 and JAK3) for the treatment of rheumatoid arthritis [17]. It was not until 5 years later that approval was granted by the European Medicines Agency (EMA). Interestingly, approval of oclacitinib (selective JAK1) for AD in dogs [18] occurred as early as 2013, followed by approvals of baricitinib (JAK1/JAK2) and upadacitinib (JAK1) for rheumatologic indications. In November 2020, baricitinib became the first JAK inhibitor approved by the EMA for AD in humans [19].

Several topical as well as systemic JAK inhibitors are currently in clinical trials for use in AD (Table 1).

## Topical JAK inhibitors

Topical therapy is the most important component of AD therapy [20, 21, 22]. In terms of anti-inflammatory therapy, glucocorticosteroids and calcineurin inhibitors (pimecrolimus, tacrolimus) are currently available. The potential long-term consequences of topical glucocorticosteroid therapy often lead to a negative attitude of patients towards this form of therapy (“steroid phobia”). Calcineurin inhibitors are used especially in sensitive areas [20, 21], but occasionally the effect is not sufficient for adequate control of the disease. Likewise, the systemic therapeutics in particular bring potential adverse drug effects, so additional, more effective, topical therapies with good safety profiles are desirable.

There are several topical formulations of JAK inhibitors in clinical testing. In addition, there are compounds with dual actions that are in preclinical stages for topical use in AD, such as a JAK3/tropomyosin receptor kinase A (TrkA) inhibitor, or have been used in early clinical trials such as the JAK/SYK inhibitor cerdulatinib (see below). Last year, delgocitinib (Corectim, Japan Tobacco Inc., Tokyo, Japan) was approved as the first topical JAK inhibitor in Japan for the indication “atopic dermatitis”.

### Delgocitinib (JTE-052)

Delgocitinib, initially named JTE-052, is a JAK inhibitor with inhibitory effects on JAK1, JAK2, JAK3, and TYK2 (Figure 1). In the phase I study published in 2018, a total of 66 healthy volunteers and AD patients were enrolled to evaluate safety, tolerability, and pharmacokinetics [23]. Topical testing with 1% and 3% ointment and photo patch testing were performed. Overall, there was good tolerability with isolated lymphocytopenias. The following phase II study evaluated 0.25%, 0.5%, 1%, and 3% concentrations compared to placebo and 0.1% tacrolimus ointment in 327 AD patients [24]. The primary endpoint was improvement in the modified Eczema Area Severity Index (mEASI) after 4 weeks of twice-daily application. “Modified” to the EASI score in this study was that the head and neck region was excluded in the calculation. The ointment was applied to all affected regions except scalp, hands, and feet. The 0.5% and 1% groups showed an approximate 50% reduction in mEASI. The 3% group showed a reduction of 72.9% and the tacrolimus group 62%. Interestingly, a reduction in pruritus was measured a few hours after application. Recently, JAK1 has been shown to be involved in neuronal mediation of pruritus via the IL-4R [25], which seems to possibly contribute to this rapid effect on pruritus in AD. Furthermore, a phase II study investigated the effect as well as safety in children aged 2 – 15 years [26], showing comparable effects without serious adverse drug reactions. In a double-blind, vehicle-controlled phase III study, delgocitinib 0.5% ointment was evaluated in patients with moderate-to-severe AD [27]. A total of 158 patients aged 16 years and older participated. 106 patients applied the drug and 52 patients applied the vehicle twice daily for 4 weeks. As in the phase II study, the primary endpoint was the change in mEASI after 4 weeks. The reduction in mEASI was significantly greater in the active-agent group (–44.3% vs. 1.7%). Furthermore, 51.9% achieved an at least 50% improvement in mEASI (mEASI-50) with delgocitinib compared with 11.5% in the vehicle group. Likewise, pruritus was shown to be significantly reduced after only 1 day. In a 24-week extension phase, all patients received delgocitinib. After 24 weeks of therapy, a mEASI-50 was 95% and a mEASI-75 was 49%. Three patients developed del­gocitinib-associated eczema herpeticum during the study. Lymphopenias were not reported.

In January 2020, delgocitinib received approval in Japan as a 0.5% ointment (Corectim), making it the first approved topical JAK inhibitor worldwide [28].

### Ruxolitinib

Ruxolitinib was the first approved systemic JAK inhibitor. As a JAK1 and JAK2 inhibitor, topical application is also currently being investigated (Figure 1). Kim et al. [29] published the associated phase II study in 2020. Ruxolitinib was evaluated as a cream at four different concentrations (0.15% daily, 0.5%, 1.5%, or 1.5% twice daily) against triamcinolone 0.1% cream and placebo in 252 patients for 4 weeks. The 1.5% twice-daily dosage resulted in a 71.6% reduction in EASI after 4 weeks. A good safety profile was seen. The results on improvement of pruritus as well as quality of life were considered in a separate publication [30]. 42.5% of AD patients with the highest dosage (vs. 13.6% vehicle and 20.5% triamcolon group) showed already after 36 hours the so-called “minimal clinically important difference” of an improvement of pruritus measured by the numerical rating scale of 2 points. The study was also conducted in pediatric patients, but no data have been published yet. Recently, the effects of a 1.5% ruxolitinib cream were also analyzed in an IL-33 transgenic mouse model [31] showing downregulation of Th2 associated proteins such as IL-33 and IL-4R by the application of the cream. Also a lower expression of molecules of the interferon signaling pathway like IFITM3 (interferon-induced transmembrane protein 3) was shown. In two phase III trials with a total of 1,249 patients, ruxolitinib 0.75%, 1.5% or vehicle was applied for 8 weeks [32]. Overall, approximately half of the patients in the active-agent groups achieved near complete or complete cure. Recently published pharmacokinetic studies in patients with an affected area of up to 20% give no indication of plasma concentrations of ruxolitinib that would result in systemic adverse drug effects [33].

### Tofacitinib

JAK1 and JAK3 are selectively and irreversibly inhibited by tofacitinib. In vitro studies showed that this specifically blocks Th1-, Th2- as well as Th17-associated cytokines and the activation of STAT1, STAT3, STAT4, STAT5, and STAT6 [34, 35]. In March 2017, approval was granted as a systemic medication for rheumatoid arthritis. For a topical application, a phase IIa study was published in 2016 evaluating tofacitinib 2% ointment in a randomized, double-blind, vehicle-controlled trial in 69 patients with mild-to-moderate AD [36]. After twice-daily application for 4 weeks, there was an 81.7% improvement in EASI compared with 29.9% with vehicle application. There were 12 adverse events in the tofacitinib group (in 11 patients) compared with 26 adverse events (in 19 patients) in the vehicle group. None of the adverse events were considered serious.

This is the first published successful proof-of-concept study with a topical JAK inhibitor in AD. However, at this time, no further studies have been registered and the compound is unlikely to be further developed clinically in this formulation.

### Cerdulatinib (DMVT-502)

Cerdulatinib, also known as DMVT-502, is a dual JAK and SYK inhibitor that has demonstrated efficacy in lymphoma as an oral therapy. Spleen tyrosine kinase (SYK) is involved in mediating proinflammatory signaling, including via the dectin-1 receptor, which is implicated in the inflammatory processes of AD [37]. Inhibition accordingly leads to a reduction in the release of proinflammatory cytokines such as IL-1.

First, in an AD mouse model, daily application of 0.18% and 0.37% cerdulatinib gel (initially named RVT-502) was shown to reduce inflammation in the skin as well as epidermal hyperplasia. In 2021, Piscitelli et al. [38] published a double-blind, monocentric, vehicle-controlled phase Ib study evaluating cerdulatinib (DMVT-502) as a 0.37% gel twice daily for 14 days in a total of 10 mildly to moderately affected AD patients (8 cerdulatinib vs. 2 vehicle). After 14 days, there was a significant 2.6-point reduction in EASI. Epidermal thickening and dendritic cells were reduced in skin biopsies of patients using cerdulatinib. Eight of ten patients in the study reported a total of 35 adverse events, with cephalgia being the most common adverse event.

Larger phase II studies are needed to elucidate the safety as well as the effect of cerdulatinib.

### SNA-125

SNA-125 is a topical JAK3 and tropomyosin receptor kinase A (TrkA) inhibitor. Inhibition of JAK3 results in inhibition of IL-4 and TSLP signaling, among others. TrkA represents the high-affinity receptor for NGF (nerve growth factor), which is elevated in AD skin and plays a role in neuroinflammation and pruritus. Its application has already been illuminated in a psoriasis mouse model. Phase I and II studies seem to be planned for AD [8].

## Systemic JAK inhibitors

JAK inhibitors, unlike monoclonal antibodies such as dupilumab, can be administered orally due to their chemical structure and size. Taking baricitinib, which is approved for AD, as an example, a bioavailability of 79% shows a low first-pass effect. Elimination of JAK inhibitors occurs either primarily by renal excretion (including baricitinib, upadacitinib) or metabolization (including ruxolitinib, tofacitinib). The JAK inhibitors with hepatic metabolization show a short half-life of mostly ~ 3 hours, so that twice-daily administration is necessary. JAK inhibitors with primary renal elimination show longer half-lives, mostly between 9 and 14 hours so that a single administration per day is sufficient.

### Baricitinib

In addition to dupilumab, another “first-line” systemic therapy, baricitinib, has been approved. On September 18, 2020, the EMA granted an extension of the marketing authorization for baricitinib for the treatment of adult patients with moderate to severe AD [39]. Baricitinib is a JAK1 and JAK2 inhibitor that is used in rheumatology for the treatment of rheumatoid arthritis, but here usually in combination with methotrexate [19].

The efficacy of this inhibitor for the treatment of moderate to severe AD was demonstrated, among others, in a phase II study of baricitinib in combination with topical glucocorticosteroids. After 16 weeks, based on an EASI-50, there were significantly better results with 4 mg baricitinib per day (61%) compared to the placebo group (37%) [40].

Two independent, multicenter, double-blind phase III studies (BREEZE-AD1 and BREEZE-AD2) followed, which were published in January 2020 [41]. The aim of the studies was to evaluate the efficacy of the inhibitor in patients with moderate-to-severe AD and inadequate response to adequate topical therapy. 1,239 patients received baricitinib at varying doses (1 mg, 2 mg, 4 mg) or placebo for 16 weeks. The primary endpoint was defined by an Investigator’s Global Assessment (IGA) of 0 or 1, equivalent to complete or near-complete healing, with an improvement of 2 or more points with respect to baseline visit scores. In summary, more patients on 4 mg or 2 mg per day reached the primary endpoint than in the placebo group (for 4 mg dosing in BREEZE-AD1 16.8% placebo: 4.8%; and in BREEZE-AD2 13.8%, versus placebo: 4.5%). Other secondary endpoints included an EASI-75/-90, relief of pruritus and pain, improvement in night sleep, and an increase in quality of life. All of the above secondary endpoints were significantly improved in the 4-mg groups. In particular, rapid pruritus relief, which was significantly evident with 4 mg compared to the placebo group after only 1 week, occurred in both studies. Furthermore, a higher efficacy was shown with the 4-mg dosage compared to the 1-mg and 2-mg dosages. This resulted in the approved dosage of 4 mg. A 2-mg dosage is recommended especially for age 75 and older, recurrent infections, or renal function impairment. In another phase III study focusing on quality of life and productivity, this also showed rapid and significant improvement as measured by various scores, including the Dermatology Life Quality Index (DLQI) and Patient Benefit Index (PBI) [42].

A meta-analysis of eight studies published in 2021 signaled a good overall safety profile for this JAK inhibitor [43]. The most common adverse drug reactions included upper respiratory tract and herpes simplex virus (HSV) infections, which occurred significantly more often in the active-agent group, regardless of dose. Severe HSV infections, also known as eczema herpeticum (EH), occurred in 4 of 743 (0.5%) patients in the placebo group and in 32 of 2,531 (1.4%) patients in the baricitinib groups. Looking at the different baricitinib doses, the increased risk for EH was seen only in the 4-mg group. Since one an EH in the last year or two or more EHs in the history was an exclusion criterion in the pivotal studies, registry data is now needed to determine the risk of HSV infections in different severities compared to other anti-inflammatory therapy in AD in the “real-life” scenario.

Furthermore, the pivotal studies showed laboratory elevations for creatinine kinase (4 mg baricitinib: 23.8%, 2 mg baricitinib: 19.3% versus placebo: 10.3%) that were clinically asymptomatic, as well as an increase in blood lipid levels. Previous studies have demonstrated an increased risk of thromboembolic events with JAK inhibitors [44], which was not observed in the AD patient population for baricitinib. Advantageous aspects of therapy with baricitinib include oral availability and rapid onset of action, including rapid relief of pruritus. Additionally, there is now an alternative to dupilumab that can be administered in case of loss of efficacy or intolerance against the therapeutic antibody.

### Upadacitinib

Upadacitinib is a second-generation oral selective JAK-1 inhibitor that is already approved for the treatment of rheumatoid arthritis [45].

A phase IIb study also showed good, dose-dependent results for the treatment of moderate-to-severe AD [46]. In the multicenter, double-blind, placebo-controlled study, 164 patients received monotherapy with upadacitinib at varying doses (7.5 mg, 15 mg, 30 mg), or placebo for 16 weeks. The primary endpoint was percent improvement in EASI score. Results showed a dose-dependent improvement in EASI compared to the placebo group (7.5 mg: 39%; 15 mg: 62%; 30 mg: 74%; vs. placebo 23%). There was also significant dose-independent improvement in pruritus after only 1 week. Upper respiratory tract infections were reported as adverse events. Furthermore, worsening of AD or cases of acne were described occasionally in the study. In the phase III studies published in 2021, two identical studies with a total of 847 and 836 AD patients aged 12 – 75 years, respectively, (Measure Up 1 and 2 study) were presented [47]. Patients received either upadacitinib 15 mg, 30 mg, or placebo once daily. After 16 weeks, the co-primary endpoint of an EASI-75 and an IGA of 0 or 1 was considered. An EASI-75 was achieved by 60 – 80% of patients in the active-agent groups vs. 13 – 16% in the placebo groups, depending on dose and study. There were increased frequencies of acneiform skin lesions and infections in the active-agent groups, especially in the 30-mg dose group. Another phase III study evaluated the two doses with concomitant topical anti-inflammatory therapy [48]. An EASI-75 was achieved by 65% and 77% in the 15-mg and 30-mg upadacitinib groups, respectively, and 26% in the placebo group. The adverse event profile was comparable to the other phase III studies. A phase IIIb study was also completed in December 2020 that directly compared dupilumab 300 mg every 2 weeks and upadacitinib 30 mg per day. In the associated publication, the authors report that 71% of patients in the upadacitinib group and 61% in the dupilumab group met the primary endpoint of an EASI-75 at 16 weeks, which was a significant difference between the two groups, with the higher upadacitinib dose of 30 mg used here [49].

Currently, several studies are being conducted for the use of upadacitinib in children. Among others, a current open-label study with upadacitinib for 6-month to 12-year-old patients with severe AD is analyzing safety and pharmacokinetics [50]. In October 2020, the manufacturer has already submitted a marketing authorization application to the EMA and FDA for the treatment of moderate-to-severe AD [51]. On June 24, the Committee for Medicinal Products for Human Use (CHMP) issued a positive opinion regarding upadacitinib for the treatment of moderate-to-severe AD. On August 24, 2021, the EMA approved upadacitinib for the treatment of moderate to severe AD in adults and adolescents 12 years and older who are candidates for systemic therapy. Consequently, upadacitinib represents the first JAK inhibitor with approval for therapy also in adolescents. For patients aged 18 – 64 years, either 15 mg or 30 mg can be used, depending on the severity of the disease; for adolescents and adults aged 65 years or older, 15 mg is recommended.

### Abrocitinib (PF04965842)

Abrocitinib belongs to the group of selective JAK1 inhibitors. 267 patients between 18 and 75 years of age were enrolled in a randomized, placebo-controlled phase II study [52]. The effect of the 10-mg, 30-mg, 100-mg, and 200-mg once daily doses were studied based on the primary endpoint of an IGA of 0 or 1 at 12 weeks. There were significant differences in the 100-mg and 200-mg groups compared to the placebo group. Surprisingly, there was a dose-dependent decrease in platelets in the first 4 weeks. Since pathophysiologically thrombocytopenia is attributed to JAK2 inhibitors (Figure 1), due to mediation of thrombopoietin (TPO) signaling via JAK2, and abrocitinib at the doses used should relatively selectively inhibit JAK1, this adverse drug effect was further investigated by the manufacturer in mechanistic models. It is hypothesized that IL-6 both stimulates TPO production via JAK1-coupled receptors and directly affects platelet formation [53, 54]. Two phase III trials were published in 2020 (JADE MONO-1 and 2). The “JADE MONO-1” trial, a multicenter, double-blind, placebo-controlled study enrolled 387 patients with moderate-to-severe AD [55]. Patients were aged 12 years and older. Two dose groups (200 mg vs. 100 mg) and a placebo group were studied with respect to the co-primary endpoints of achieving an IGA of 0 or 1 and at least 75% improvement in EASI (EASI-75). 63% of patients in the 200-mg group, 40% in the 100-mg group, and 12% in the placebo group demonstrated an EASI-75 at 12 weeks. 44% using the higher dose achieved almost complete or complete resolution of AD. The JADE MONO-2 study represented an identical placebo-controlled phase III trial of 391 moderately to severely affected AD patients aged 12 years and older [56]. An EASI-75 improvement was also shown to be significantly more frequent in the actively treated groups compared to the placebo group at 12 weeks in this study (61.0% vs. 44.5% vs. 10.4%). Topical glucocorticosteroids as adjunctive therapy were prohibited in both studies. In addition, as in the phase II study, there was a dose-dependent decrease in platelets. The JADE COMPARE study of 838 moderately to severely affected AD patients evaluated the two doses in comparison to dupilumab as well as placebo [57]. The primary endpoint was equivalent to that in the other phase III studies. An EASI-75 was seen in 70.3% with 200 mg of abrocitinib, 58.7% with 100 mg abrocitinib, 58.1% in the dupilumab group, and 27.1% in the placebo group. Concomitant topical glucocorticosteroid therapy was allowed.

In summary, abrocitinib shows good efficacy in the treatment of moderate-to-severe AD. There is currently a review of the approval of abrocitinib for the treatment of AD in adolescents and adults by the FDA as well as the EMA. A statement from the FDA was expected in April 2021, but has already been postponed twice.

### Tofacitinib

Tofacitinib is already approved as a JAK1 and JAK3 inhibitor for the treatment of rheumatoid arthritis, psoriatic arthritis, and ulcerative colitis. Only one small proof-of-concept study with 6 AD patients can be found in the literature. Five patients received the rheumatoid arthritis dosage (5 mg 2 × daily), and 1 patient received only 5 mg 1 × daily [58]. Follow-up after 8 – 29 weeks showed a reduction in SCORAD of two-thirds of baseline. Larger-scale trials are not found at clinicaltrials.gov, so an expansion of the approval of tofacinitib to AD does not appear to be sought.

To date, German physicians have been informed, via several so-called red hand letters, about a possible increased risk of serious adverse cardiovascular events, malignant diseases [59] as well as venous thromboembolic events and serious infections [60]. In the previously summarized studies on JAK1 and JAK2 selective inhibitors, these adverse events have not been observed to be significantly clustered in the indication “atopic dermatitis” at the doses used there so far. Whether this is due to the different patient characteristics, different reactivities of the substances, or the fact that in rheumatology JAK inhibitors, unlike in AD, are usually combined with an immunosuppressive basic therapy such as MTX, remains to be seen.

### Gusacitinib (ASN002)

Gusacitinib inhibits SYK in addition to the four JAK molecules. In a randomized, double-blind, placebo-controlled phase Ib study, 36 adult AD patients were treated with 20 mg, 40 mg, or 80 mg gusacitinib or placebo for 28 days [61]. An EASI-50 was achieved significantly more often in the 40-mg and 80-mg groups (20 mg: 20%, 40 mg: 100%, 80 mg 83%, placebo: 22%). Molecular serum analyses showed a reduction in, among others, Th2 cytokines and chemokines such as IL-13, CCL13, and CCL17. Complementary transcriptome analyses showed suppression of the Th2 (Il-4R, CCL13/17/18/22/26) as well as Th1 (IFN, among others) and Th17/22 axes, particularly in the 40-mg and 80-mg groups [62]. A phase IIb study with 250 planned patients has already completed enrollment in August 2019. The linked extension study has been terminated and not fully completed, according to clinicaltrials.gov. Results of the trials have not been published to date. Currently, gusacitinib is also being studied for chronic hand eczema in clinical trials. Recent press releases reported a “fast track” submission of gusacitinib to the FDA for the indication of chronic hand eczema.

It remains to be seen in what form gusacitinib will be further investigated for AD. It is possible that it will be primarily pursued for the indication of chronic hand eczema.

## Conclusion

JAK inhibitors are important new therapeutic options in the treatment of AD. Topical application in particular is expected to play an important role in new therapeutic concepts. The systemic JAK inhibitors show a rapid efficacy with also rapid improvement of pruritus. The tolerability is good in patients with AD based on the registration studies of the substances baricitinib, upadacitinib, and abrocitinib, but further data from patient registries such as TREATgermany are needed to assess this even better.

## Funding

None.

## Conflict of interest

S. Traidl received consultancy fees from Leo Pharma, Lilly and La Roche-Posay outside the submitted work.

S. Freimooser has nothing to disclose.

T. Werfel is Co-Principal Investigator of the German Atopic Dermatitis Registry TREATgermany. TW has received honoraria for lectures or scientific advice on atopic dermatitis from AbbVie, Almirall, Galderma, Jans-sen/JNJ, Leo Pharma, Leti, Lilly, Novartis, Pfizer, and Regeneron/Sanofi.

**Table 1.** The different topical and systemic JAK inhibitors with the different doses, dosing frequencies and current phase in clinical trials.

Active ingredient	Designation	Trade name	Target molecule	Application	Formulation	Dose	Frequency	Study phase	References
Delgocitinib	JTE-052	Corectim^®^	JAK1, JAK2, JAK3, TYK2	topical	Ointment	0,5%	2 × daily	Approved	[23, 24, 26, 27]
Ruxolitinib			JAK1, JAK2	topical	Cream	0,15%, 0,5%, 1,5%	1 – 2 × daily	Phase III	[29, 30, 32]
Tofacitinib			JAK1, JAK3	topical	Ointment	2%	2 × daily	Phase IIa	[36]
Cerdulatinib	DMVT-502/RVT-502		JAK, SYK	topical	Gel	0,37%	2 × daily	Phase Ib	[38]
SNA-125			JAK3, TrkA	topical				Planned	
Tofacitinib	CP-690,550	Xeljanz^®^	JAK1, JAK3	oral	Tablet	5 mg	2 × daily	Proof-of-concept	[58]
Gusacitinib	ASN-002		JAK1	oral	Tablet	20 mg, 40 mg, 80 mg	1 × daily	Phase Ib	[61]
Abrocitinib	PF -04965842		JAK1	oral	Tablet	100 mg, 200 mg	1 × daily	Phase III	[52, 55, 56, 57]
Upadacitinib	ABT-494	Rinvoq^®^	JAK1	oral	Tablet	7.5 mg, 15 mg, 30 mg	1 × daily	Phase III	[47, 48]
Baricitinib		Olumiant^®^	JAK1, JAK2	oral	Tablet	4 mg (2 mg)	1 × daily	approved	[41, 42, 43]

JAK = Janus kinase; SYK = Spleen tyrosine kinase; TrkA = Tropomyosin receptor kinase A.
